# An Overview on Fatigue of High-Entropy Alloys

**DOI:** 10.3390/ma16247552

**Published:** 2023-12-07

**Authors:** Junchao Hu, Xue Li, Qiuchen Zhao, Yangrui Chen, Kun Yang, Qingyuan Wang

**Affiliations:** 1 School of Mechanical Engineering, Institute for Advanced Study, Chengdu University, Chengdu 610106, China; cdu_hujunchao@126.com (J.H.); zhaoqiuchen@stu.cdu.edu.cn (Q.Z.); chenyangrui2003@163.com (Y.C.); 2 School of Architecture and Civil Engineering, Chengdu University, Chengdu 610106, China; lixuezi89@163.com; 3 Failure Mechanics and Engineering Disaster Prevention and Mitigation Key Laboratory of Sichuan Province, Sichuan University, Chengdu 610065, China; 4 Key Laboratory of Deep Earth Science and Engineering, Sichuan University, Ministry of Education, Chengdu 610065, China

**Keywords:** high-entropy alloy, fatigue crack initiation, fatigue crack growth, fatigue life prediction model, fatigue property

## Abstract

Due to their distinct physical, chemical, and mechanical features, high-entropy alloys have significantly broadened the possibilities of designing metal materials, and are anticipated to hold a crucial position in key engineering domains such as aviation and aerospace. The fatigue performance of high-entropy alloys is a crucial aspect in assessing their applicability as a structural material with immense potential. This paper provides an overview of fatigue experiments conducted on high-entropy alloys in the past two decades, focusing on crack initiation behavior, crack propagation modes, and fatigue life prediction models.

## 1. Introduction

With the rapid development of the economy and society, long service life and high reliability have become the ultimate goal and urgent demand for major projects and cutting-edge equipment. When materials or equipment are subjected to repeated cyclic loading, mechanical fatigue occurs. This can result in more severe damage to the equipment and even lead to catastrophic failure, potentially resulting in fatal consequences [1,2]. For a significant period, the economic losses stemming from fatigue failure, along with the inherent threats to human life and property, have remained impossible to overlook [3]. Fatigue failure remains one of the leading culprits behind mechanical part failures, accounting for over 80% of such instances [4]. Because fatigue damage does not exhibit noticeable deformation, it often leads to significant accidents. To mitigate this risk, components subjected to cyclic loads should be made from materials possessing high fatigue strength, examples of such components include shafts, gears, bearings, blades, springs, and more [5,6]. Therefore, many scholars have been committed to studying the fatigue behaviors of various materials and designing excellent anti-fatigue structural materials.

High-entropy alloys defy the traditional approach to alloy design, which usually involves incorporating small amounts of additional elements into a base alloy composed of one or two primary constituents. Instead, these alloys are composed of five or more elements in specific proportions, resulting in a single solid solution alloy with distinct atomic structure characteristics [7,8]. In the past decade, the development of material research has led to the modification of the definition of high-entropy alloys. The main element elements are composed of four or more alloying elements. The ratio of the constituent elements can be non-equiatomic ratio, and the phase structure is a two-phase or multi-phase complex solid solution alloy. The adjustment of the ratio of main elements and constituent elements is beneficial to the formation of not only single-phase solid solution structures but also two-phase eutectic and multi-phase structures of intermetallic or amorphous compounds in high-entropy alloys. High-entropy alloys, according to different literature sources [9], are classified in various ways. Although they consist of multiple elements, they usually exhibit a relatively simple phase structure after solidification, commonly forming a solid solution with Face Center Cubic (FCC), Body Center Cubic (BCC), or hexagonal close-packed (HCP) structures. In high-entropy alloys, the formation of an amorphous phase is also possible [10,11]. Moreover, the characteristics of the various principal components result in high mixing entropy in thermodynamics, severe lattice distortion in dynamics, sluggish diffusion effect, and cocktail effect in performance [9,12,13,14]. High-entropy alloys have excellent mechanical properties and corrosion resistance, they are a structural material with great application potential and can be used in construction and mechanical engineering. Because of their high temperature strength, corrosion resistance, and low density, high-entropy alloys can be used in the aerospace industry to manufacture key components such as turbine engine blades, combustion chambers, and structural components. The application of high-entropy alloys in chemical reaction vessels and catalysts is being extensively studied. They have good corrosion resistance and high temperature resistance, and can withstand extreme reaction environments. These properties provide high-entropy alloys with superior mechanical properties compared to other alloys, including high fracture toughness, and high fatigue resistance [15,16,17]. In the present era, marked by a significant need for advanced materials, high-entropy alloys are emerging as innovative high-performance structural materials. Consequently, high-entropy alloys are progressively gaining prominence as focal points of research in the realm of solid mechanics and materials science [15,18,19].

As a structural material, high-entropy alloys must have sufficient fatigue resistance to be truly applied in the engineering field. Although the basic mechanical properties (such as strength) of high-entropy alloys have been extensively studied, there are relatively few studies on their ultra-high cycle and high cycle fatigue behavior. As a structural material with great application potential, the long-life fatigue behavior and failure mechanism of high-entropy alloys are still a field worthy of further exploration. The paper reviews recent research on the fatigue behavior of high-entropy alloys. It examines the mechanism of fatigue crack initiation, discusses the influence of microstructure and temperature on fatigue crack propagation characteristics, and analyzes prediction models for fatigue life. Lastly, this review offers some insights and summaries regarding the advancement of high-entropy alloys in the field of fatigue.

## 2. Fatigue Crack Initiation Mechanism

During cyclic loading, metal materials deform and create slips. These slips eventually combine, forming wider and more concentrated slip bands called persistent slip bands (PSBs). These PSBs penetrate deeper into the surface and lead to stress localization. As a result, fatigue cracks initiate at the PSBs, grain boundaries, vacancies, and surface inclusions. In the fields of high cycle fatigue (HCF) and very high cycle fatigue (VHCF), fatigue failure is primarily influenced by fatigue crack initiation, which accounts for a significant portion of the overall fatigue life. Consequently, it is crucial to understand the mechanisms behind slip formation and stress localization to improve the fatigue life of metal materials [20]. In polycrystalline alloys, cyclic loading leads to the accumulation of slip, which affects the microstructure and mechanical properties of the material. When slip is the dominant mechanism, it can cause strain localization and initiate cracks due to plastic deformation [21]. Therefore, this section focuses on reviewing the mechanisms of crack initiation and their relationship with microstructure in high-entropy alloys.

In the study by Suzuki et al. [22], the initiation and propagation of fatigue cracks driven by slip planes were systematically investigated. The researchers believed that the essential characteristics of slip often determine the initiation process of fatigue cracks. In their experiments, equiatomic CrCoMnFeNi alloy was used to demonstrate the behavior of crack initiation and propagation in the field of high cycle fatigue. The performance of CrMnFeCoNi alloy and Fe-20Cr-14Ni alloy (ASS) was compared, and it was found that there was little difference in grain size, with the CrCoMnFeNi alloy being 41 μm and the Fe-20Cr-14Ni alloy being 38 μm. The stacking fault energy (SFE) of Fe-20Cr-14Ni alloy and CrCoMnFeNi alloy was 35 mJ^−2^ and 30 mJ^−2^, respectively. The researchers carried out a tensile test at room temperature with an initial strain rate of 10^−4^ S^−1^ along the rolling direction, they also conducted a rotating bending fatigue test at room temperature with a loading frequency of 30 Hz and stress ratio *R* of −1. The authors focused on clarifying the effect of high dislocation planarity on fatigue crack initiation and small crack propagation.

**Figure 1:**
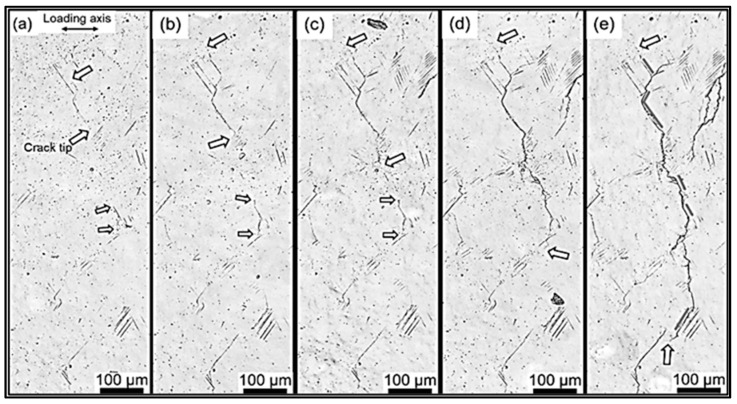
Microscopic characterization of the CrMnFeCoNi alloy with different load cycles under 270 MPa, (**a**) 4 × 10^5^ cycles, (**b**) 7.5 × 10^5^ cycles, (**c**) 9.5 × 10^5^ cycles, (**d**) 1.0 × 10^6^ cycles, (**e**) 1.05 × 10^6^ cycles, the white arrow represents the crack tip [22].

Multiple cracks can be seen in Figure 1a–e, and a high-plane dislocation array is also visible around the crack initiation point. The highly dense planar dislocation arrays surrounding the crack initiation site can be viewed as lattice defects that exist in the crystal structure and induce changes in the mechanical properties of materials. During crack propagation, the presence of these dislocation arrays imposes constraints on the crack growth path, while the lattice deformation around the dislocations also exerts a certain influence on crack propagation. Consequently, these dislocation arrays eventually cause the cracks to gradually merge, thereby forming longer crack paths. Such observations possess crucial significance in our comprehension of material fracture behavior as well as in the pursuit of mechanisms to resist crack propagation in materials.

**Figure 2:**
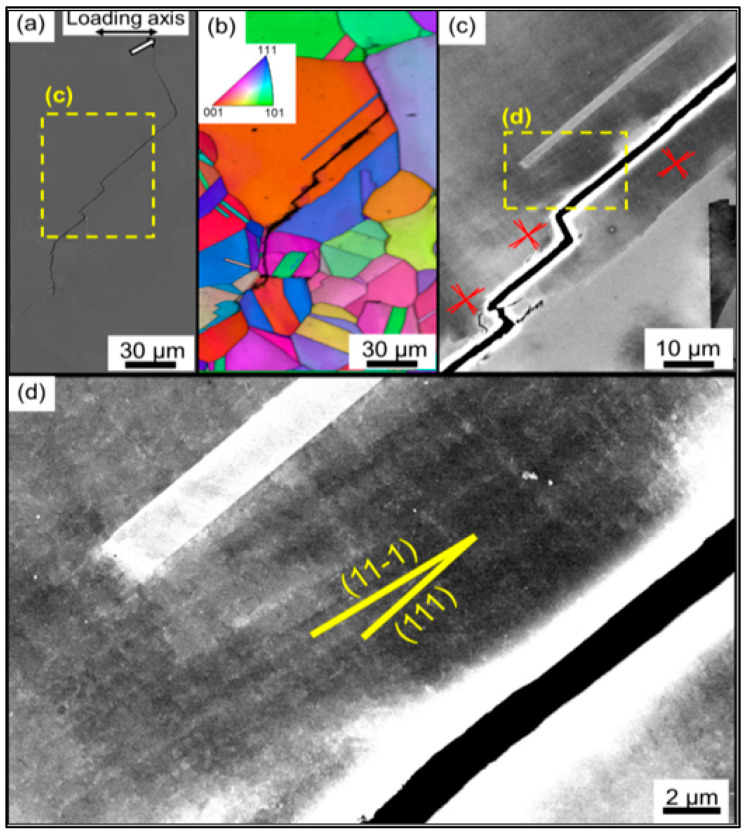
Microscopic analysis of fatigue cracks. (**a**) The macrograph of a microcrack near the main crack, where the white arrow represents the crack tip. (**b**) The inverse pole figure of the main crack. (**c**,**d**) is the amplified Electron Channeling Contrast image from the crack initiation point, the red line represents the slip plane traces, yellow lines represent two slip plane traces [22].

The crack initiation in the CrMnFeCoNi alloy was observed on the {111} slip plane and {11-1} slip plane, as depicted in Figure 2d. Once slip deformation occurs, the accumulation of dislocations on a single slip plane leads to the development of slip surfaces and subsequently triggers the initiation of fatigue cracks [23]. In high-entropy alloys, multiple slip planes exist. Activation of the {111} slip plane indicates the initiation of grain boundary sliding within the lattice [24]. However, during the sliding process, the grain boundary may encounter certain obstacles. If the obstruction at the grain boundary is significant, the slip plane may be forced to shift to a different direction, specifically activating the {11-1} slip plane. Therefore, crack initiation in CrMnFeCoNi alloys is a consequence of the combined action of {111} and {11-1} slip planes.

In high-entropy alloys, the presence of high-entropy configurations leads to a higher likelihood of dislocation formation along parallel slip planes, resulting in slip localization. When stress is applied to the material, dislocations move along these slip planes [25]. As slip progresses, the interactions between these slip planes can lead to their cracking, which is known as slip plane cracking. Given the presence of multiple slip planes in high-entropy alloys, the interactions and influences between them play a significant role in the generation of microcracks and alterations in crack propagation paths. Overall, understanding these mechanisms is crucial for predicting and controlling the mechanical behavior of high-entropy alloys.

In the study conducted by Feng Rui et al. [26], the fatigue performance of Al_0.5_CoCrFeNi was examined. It was discovered that the fatigue performance of the Al_0.5_CoCrFeNi alloy can be improved through the ductile transformation of multicomponent B2 precipitates. The cyclic deformation mechanism was explained through various techniques, including real-time in situ neutron diffraction, transmission electron microscopy, crystal-plasticity, and Monte Carlo simulations. The observed cyclic mechanisms included slip, precipitation strengthening, deformation twinning, and reversible martensitic transformation. The main focus of our review is on the crack initiation mechanism, which is caused by local stress concentration at the boundaries during non-uniform deformation between the hard B2 phase and soft FCC phase. In the specific experiments, the Al_0.5_CoCrFeNi has an FCC matrix and multi-component B2 strengthening precipitates. The B2 precipitation phase presents three different hierarchical morphologies, which are the fine and dense block, needle-like, and coarse band at the grain boundary of the FCC phase [27,28]. The FCC matrix and B2 precipitation phase satisfy the Kurdjumov–Sachs (K-S) crystallographic relationship. The fatigue fracture of the Al_0.5_CoCrFeNi alloy was microscopically characterized after a monotonic uniaxial tensile test, different strain amplitudes fatigue test, and atomic median diffraction test. The purpose of microscopic characterization is to observe the microstructure evolution during cyclic deformation and to discuss the crack initiation mechanism of Al_0.5_CoCrFeNi alloy.

**Figure 3:**
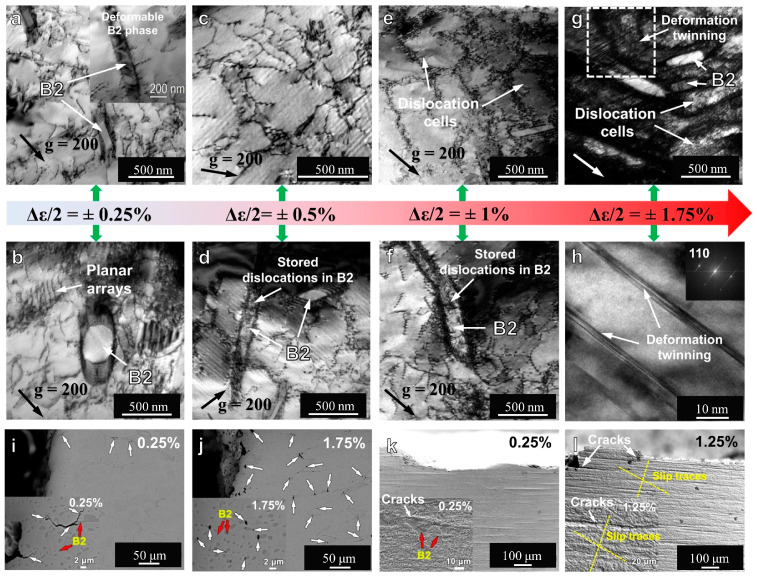
Microstructure evolution diagrams under different strain amplitudes, (**a**–**h**) shows the transmission electron microscope (TEM) bright-filed (BF) images under the strain amplitudes of ±0.25%, ±0.5%, ±1%, and ±1.75%, respectively. (**i**–**l**) shows the scanning electron competitive images of the fracture specimens under the strain amplitudes of ±0.25%, ±1.25%, and ±1.75%, respectively, showing the crack nucleation characteristics under high and low strain amplitudes. The largest red gradient arrow represents different strain amplitudes, and the strain amplitude increases gradually from left to right. The green arrow represents the microstructure characterization results under different strain amplitudes. The meaning represented by the arrows of other colors has been marked in the figure [26].

It can be found that slip is the dominant mechanism of the micro deformation mechanism from low strain amplitude (Figure 3a–h) below 1%, and few micro-cracks initiate at low strain amplitude (0.25%). When the strain amplitude increases to 1%, due to the active cross slip in the FCC matrix, dislocation cells are formed, but no deformation twins are found. When the strain amplitude was further increased to 1.75%, the elongated dislocation cells separated by denser and severely tangled dislocations and the deformation twins generated in the more severely tangled region (Figure 3g,h) were observed. The results show that these microcracks mainly initiate and propagate near the larger B2 precipitates. This is due to the long incoherent interface of the precipitates, which cannot effectively adjust the stress concentration [27,28,29,30]. With cyclic loading, plastic deformation initially appears in the soft FCC matrix, leading to the multiplication and recombination of dislocation substructures within the matrix. Meanwhile, hard B2 precipitates act as hindrances and resistance to plastic deformation. With the further movement of hindered dislocations, the development of internal interphase stress promotes cyclic strain hardening. The plastic deformation gradually extends to the hard B2 phase as the cyclic number and strain increase, resulting in a large number of dislocations in the B2 phase and severe plastic deformation of the B2 phase at low strain amplitude. Moreover, under all the strain amplitudes, due to the strain incompatibility between FCC matrix and B2 phase, dense dislocations were accumulated around the B2 precipitates. Therefore, due to the difference in hardness and mechanical properties between the hard B2 phase and the soft FCC phase, inhomogeneous deformation occurs during cyclic loading [31]. This inhomogeneous deformation creates local stress concentrations at the interface between the two phases, which can result in the initiation of microcracks [32].

Based on the whole crack initiation process, The local strain localization caused by slip does not lead to obvious crack initiation, while the micro-cracks mainly initiate near the banded B2 phase, and it can be observed that the crack tip becomes blunt near the fine B2 precipitates and forms an annular crack. One such phenomenon demonstrated that the ductile-transformable B2 phase improves fatigue resistance, alters the general crack initiation mechanism, and ultimately improves fatigue performance [33]. In summary, the existence of a multi-component B2 phase in high-entropy alloys increases the energy barrier for dislocation nucleation and reduces dislocation strength, thereby retarding the crack propagation rate and enhancing the material’s fatigue life. By introducing a multi-component B2 phase with ductility and transformability into high-entropy alloys, significant improvement in fatigue life can be achieved, providing new avenues and methods for the application of high-entropy alloys in engineering fields.

Kaimiao Liu et al. [34] investigated the impact of nano-precipitates on the fatigue properties of the Al_0.7_CoCrFeNi alloy with a lamellar structure. Several studies have investigated Al_x_CoCrFeNi alloys. Among these, Hemphill et al. [32] conducted a study on the fatigue behavior of cold-rolled Al_0.5_CoCrFeNi and found that fatigue failure was caused by crack initiation at the Al_2_O_3_ inclusion and the original small crack. In the study of Tang et al. [35], it was concluded that fatigue crack initiation occurs in the voids of the material resulting from rolling and annealing of the Al_0.5_CoCrFeNi alloy. In addition, Kaimiao’s article discusses the effect of persistent slip bands (PSBs) on fatigue crack initiation. Recent research has shown that PSBs play a significant role in the initiation and propagation of fatigue cracks [36,37], ultimately leading to fatigue failure in coarse-grained materials. In the study of Shukla et al. [38], the effect of PSBs on the fatigue properties of AlCoCrFeNi_2.1_ alloy was reported. Comparing the fatigue behavior of as-cast and cold-rolled annealed AlCoCrFeNi_2.1_ alloys, the as-cast AlCoCrFeNi_2.1_ alloy showed crack initiation along the boundary of the PSBs in the FCC layer. PSBs are textures that occur in materials, specifically referring to the formation and existence of slip bands caused by stress or loading [39]. PSBs can be found in various materials such as metals, ceramics, and polymers, usually forming parallel band-like structures along specific crystal directions within the material. Under normal conditions, these slip bands may appear during loading and then disappear upon unloading. However, in certain special cases, such as at high temperatures and high strain rates, PSBs may persist. Serrated crack propagation paths may be observed during cyclic loading when multiple slip systems are activated and PSBs subsequently form. The following discussion focuses on how Liu Kaimiao et al. analyzed the fatigue crack initiation mechanism of the Al_0.7_CoCrFeNi alloy under different microstructures.

According to Kaimiao Liu et al. [34], they introduced nano-sized L1_2_ precipitates into the FCC phase via low-temperature annealing. Two different microstructures were obtained through two different heat treatments. The first treatment involved homogenization at 1100 °C for 10 min followed by water cooling, referred to as the homogenization condition (AH). The second treatment involved cold rolling the homogenized alloy and annealing it at 580 °C for 24 h, referred to as the Ah + LTA condition. The fully-reversed (*R* = 1) bending fatigue test was carried out on an Al_0.7_CoCrFeNi HEA with a cyclic loading frequency of 20 Hz.

**Figure 4:**
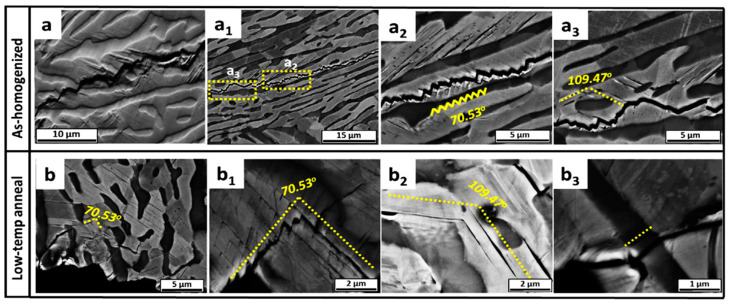
Microstructure characterization diagram of Al_0.7_CoCrFeNi alloy with two different treatments after loading. (**a**) Microscopic characterization of PSBs. (**a_1_**–**a_3_**) is the back-scattered electron image of slip band under as-homogenized conditions, (**b**,**b_1_**–**b_3_**) is the BSE image of slip band under low-temperature annealing conditions [34].

**Figure 5:**
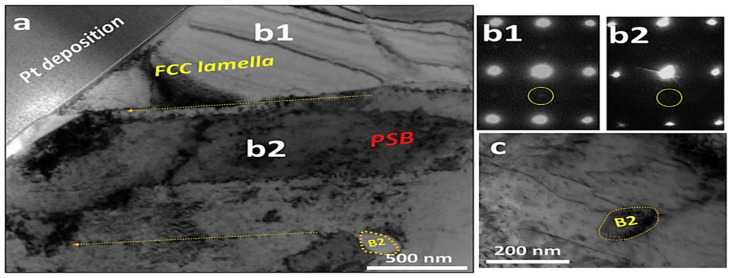
(**a**) Results of microstructure formed under AH + LTA under BFTEM, (**b1**) SADPs obtained in FCC lamellar, (**b2**) SADPs obtained in PSB, (**c**) dislocation accumulation near B2 under high-power TEM scanning, SADPs are abbreviations of selected area diffraction patterns [34].

The formation and presence of PSBs have significant effects on the mechanical properties and damage behavior of materials. They can improve the material’s ability to undergo plastic deformation and reduce stress concentration [40]. However, PSBs can also cause local degradation and damage, potentially leading to the initiation and propagation of cracks under subsequent loading [41]. In Figure 4, PSBs near the main crack can be observed, and the high-magnification SEM images show the formation of fine PSBs under two different microstructures. From Figure 4a_1_–a_3_,b_1_–b_3_, it can be concluded that in addition to stress concentration leading to crack initiation along the phase boundary, fatigue cracks initiate and propagate along PSBs. Based on the information presented in Figure 4, it also can be inferred that the angles observed among the various persistent slip bands are uniform. This indicates that a particular slip system aligned with the {111} plane has been triggered [42]. As the fatigue loading continues, plastic slip gradually moves toward the softening zone, generating a large number of slips in a particular region that produces a persistent slip band. Eventually, cracks are initiated along these persistent slip bands. From Figure 5, the superlattice corresponding to the ordered L1_2_ phase in FCC lamella containing persistent slip bands (PSBs) is reduced in the slip band, which proves that the precipitated phase is indeed sheared during cyclic loading. Moreover, there is a notable accumulation of dislocations around the fine B2 phase. These two microscopic deformations ultimately lead to the initiation of cracks. In summary, fatigue cracks initiated and propagated along the B2/FCC lamellar phase boundary, as well as the PSBs in the FCC matrix. The serrated crack propagation mode is attributed to the activation of multiple slip systems.

Gyung Tae Lee et al. [43] investigated the effect of deformation twins and dislocation cell structure on the fatigue properties of CoCrFeMnNi alloy through a rotating bending fatigue test. The dislocation cell structure and deformation twins were produced by forging CoCrFeMnNi alloys at room temperature (27 °C) and low temperature (−196 °C), respectively. After forging, it was discovered that the triple junctions of the grain boundaries formed a small number of large pores at room temperature, while in specimens at low temperature, a large number of small holes were formed at the intersection of deformation twins and grain boundaries. As a result, subsequent research and discussion mainly focused on the influence of micro-voids in different microstructures on fatigue crack initiation and fatigue performance.

To accurately compare fatigue behavior and microstructure defects between room temperature (RT) and low temperature (CT) specimens, the consistent tensile strength of the metal specimens is crucial as it significantly impacts the metal’s fatigue strength [44]. To fulfill this requirement, the researchers measured the tensile strength of the RT sample after 30% pre-stretching at room temperature and then adjusted the pre-stretching conditions according to the low temperature to make the tensile strength of the CT sample equal to that of the RT sample as much as possible. The fatigue test is based on 10^7^ cycles, the test results show that the fatigue strength deviation between the two specimens is about 80 MPa, this deviation could be attributed to microstructural variances caused by different pre-stretching temperatures. Because different microstructures will produce different crack nucleation modes, we focus on the influence of the dislocation cell structure of the RT specimen and deformation twinning of the CT specimen on fatigue behavior from the perspective of microstructure. In Lewis’ study [45], under external loading, the highest stress in metallic materials is typically observed at grain boundary triple junctions, while the second highest stress occurs at regions where grains intersect with twin boundaries. Stress concentration primarily occurs at grain boundaries, which reduces the critical energy required for crack nucleation and leads to the formation of micro-voids. According to Sangid’s article [46], it has been pointed out that under cyclic stress, due to dislocation pile-up and stress concentration, cracks preferentially nucleate near twin boundaries. The two aforementioned articles elucidate the regional and causal aspects of crack initiation mechanisms, thereby facilitating a deeper comprehension of the impact that micro-voids in various microstructures have on fatigue crack initiation and fatigue performance.

**Figure 6:**
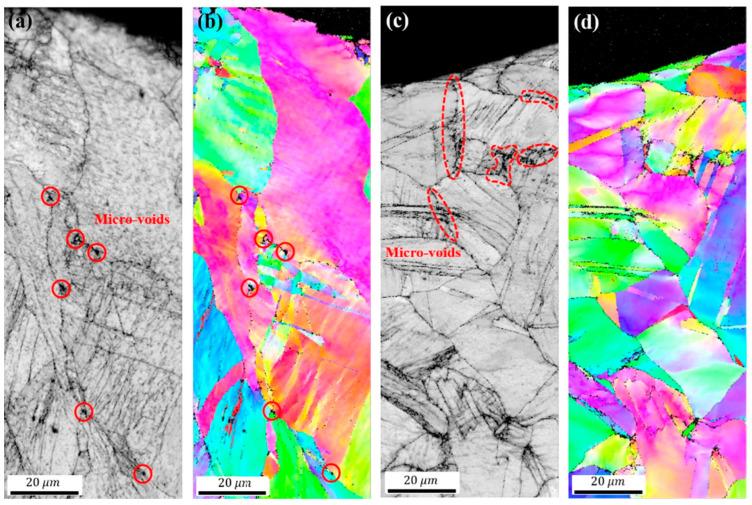
(**a**,**b**) are the EBSD scanning results of RT specimen at fatigue crack initiation site, (**a**) is EBSD IQ, (**b**) is IPF map, (**c**,**d**) are EBSD scanning results of CT specimen at fatigue crack initiation site, (**c**) is EBSD IQ, (**d**) is IPF map [43].

**Figure 7:**
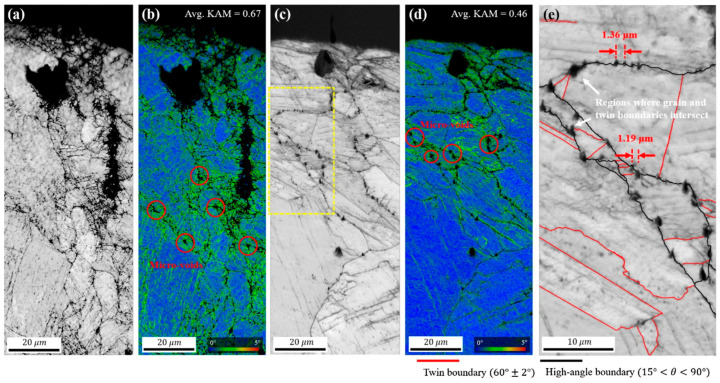
(**a**,**b**) are the EBSD scanning results of RT specimen at the fatigue crack initiation site, respectively. (**a**) is EBSD IQ, (**b**) is KAM, (**c**,**d**) are the EBSD scanning results of CT specimen at the fatigue crack initiation site, respectively. (**c**) is EBSD IQ, (**d**) is KAM, and (**e**) is the area in the yellow dotted line box of (**c**) [43].

In Figure 6, we can observe that the RT sample forms micro-voids at grain boundary triple junctions, while the CT sample forms micro-voids at grain boundaries and twin boundaries. In Figure 7, it is observed that the distance between the micro-voids in the CT sample is closer than that in the RT sample. Under the action of cyclic stress, the crack at the fatigue crack initiation site repeatedly expands and closes, forming fatigue striations. During this process, if there are voids inside the material, they act as notches, facilitating crack nucleation and allowing adjacent voids to easily connect and grow into cracks [47,48,49]. Furthermore, the size and spacing of micro-voids also play a significant role in crack initiation. Since all closely spaced micro-voids are involved in the nucleation and growth process of cracks, larger micro-void size and shorter micro-void spacing result in faster fatigue crack initiation. Therefore, CT specimens are more prone to fatigue failure than RT specimens. Consequently, analyzing the micro-morphology as a contributing factor to the initiation of material cracks is an important consideration.

## 3. Fatigue Crack Growth Behavior

Researching fatigue crack propagation is crucial for elucidating material damage and failure mechanisms, providing valuable insights for material enhancement and engineering design. Before the discussion, it is important to note that the key parameters commonly used in fatigue studies related to the topic include: the crack length (α), the number of cycles (*N*), and the stress-intensity-factor range (Δ*K*) [50]. Before conducting experiments, it is crucial to note that the growth of fatigue cracks can be roughly categorized into three stages: stage I, crack initiation; stage II, crack propagation; and stage III, instantaneous fracture. In stage I, cracks initiate and propagate along the maximum shear stress around 45 degrees, known as the short crack initiation and propagation stage. The crack will continue to propagate until it encounters obstacles, such as grain boundaries, inclusions, or pearlite areas. In stage II, the direction of crack propagation and load direction changes from 45° to 90° as the crack continues to grow. Consequently, the actual load increases, causing the stress intensity factor (*K*) to rise, which leads to slip occurring in different planes near the crack tip. During the transition from stage II to stage III, the crack tip intensity factor gradually surpasses the critical pressure intensity factor, leading to rapid crack instability and propagation. At this stage, crack propagation is controlled by the static mode of failure, which is highly sensitive to the microstructure and load-to-stress state [51]. In the follow-up discussion, the influencing factors of fatigue crack propagation will be discussed in detail.

### 3.1. Effect of Temperature on Fatigue Crack Growth Behavior

The effect of temperature on the crack growth behavior of CrMnFeCoNi alloy was investigated by Thurston et al. [52]. The CrMnFeCoNi alloy ingot was sealed in a quartz tube and subjected to hot homogenization for 48 h at 1473 K. After that, it was rotary dipped at room temperature to reduce its diameter and then recrystallized at 1073 K for 1 h. The fatigue crack growth test was conducted at 293 K and 198 K (*f* = 25 Hz). To ensure accurate test results, the experiment was carried out under low-temperature conditions using dry ice and ethanol to avoid potential thermal cycling effects. From the existing research [53], when the temperature changes from 293 K to 198 K, the ultimate tensile strength increases from 760 MPa to 925 MPa, yield strength increases from 410 MPa to 520 MPa, and ductility increases from 0.6 to 0.7. Under the premise of keeping other mechanical properties unchanged in the same temperature range [54], Young’s modulus and fracture toughness are slightly improved, respectively, from 202 GPa to 209 GPa, 217 MPa√m to 221 MPa√m. The strain strengthening index basically remained at a high level of 0.4, the trend of these high-performance indicators still exists when the temperature drops to 77 K, confirming the conclusion that the strength and ductility of high-entropy alloys are particularly strongly dependent on temperature.

**Figure 8:**
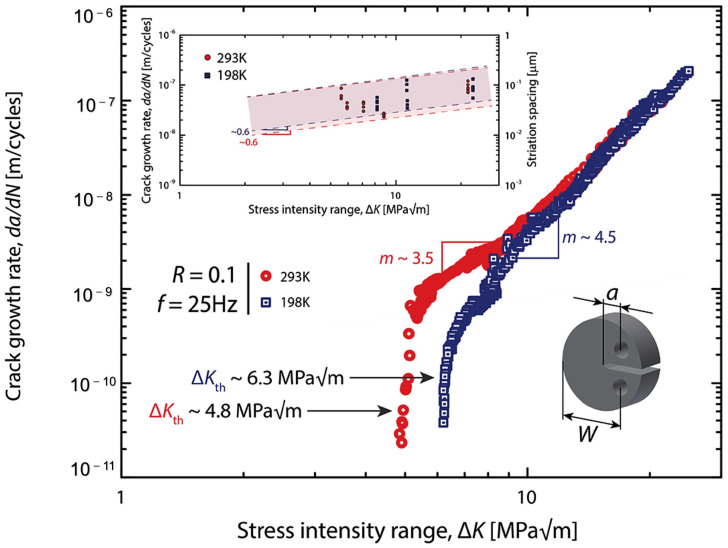
Fatigue-crack growth behavior of the CrMnFeCoNi alloy in different temperatures, the horizontal coordinate is the stress intensity range and the vertical coordinate is the fatigue crack expansion rate, samples tested on disc-shaped compact-tension (DC(T)) at a frequency of *f* = 25 Hz. The smaller, solid symbols in the inset represent the relationship between the variation of local crack-growth rates, estimated from striation spacing measurements on scanning electron microscopy images of the fracture surfaces, and Δ*K* [52].

From Figure 8, the test results have revealed a significant increase in the fatigue threshold of the alloy, by approximately 30% from 4.8 MPa to 6.3 MPa. As the temperature decreased from room temperature to a cryogenic environment (293 K to 198 K), the Paris exponent *m*, increased from 3.5 to 4.5. These findings suggest that the near-threshold growth rate and fatigue threshold Δ*K_th_* of the alloy are significantly higher in a low-temperature environment. To further investigate the impact of temperature on fatigue crack propagation [48,55,56], the fatigue crack extension experimental parameters and fatigue threshold results of several high-entropy alloys are summarized in Table 1. The comparison of the results indicates that different alloys and the same alloys at different temperatures have an impact on the final fatigue threshold and Paris coefficient [31,35,47,49,57,58,59,60,61].

**Figure 9:**
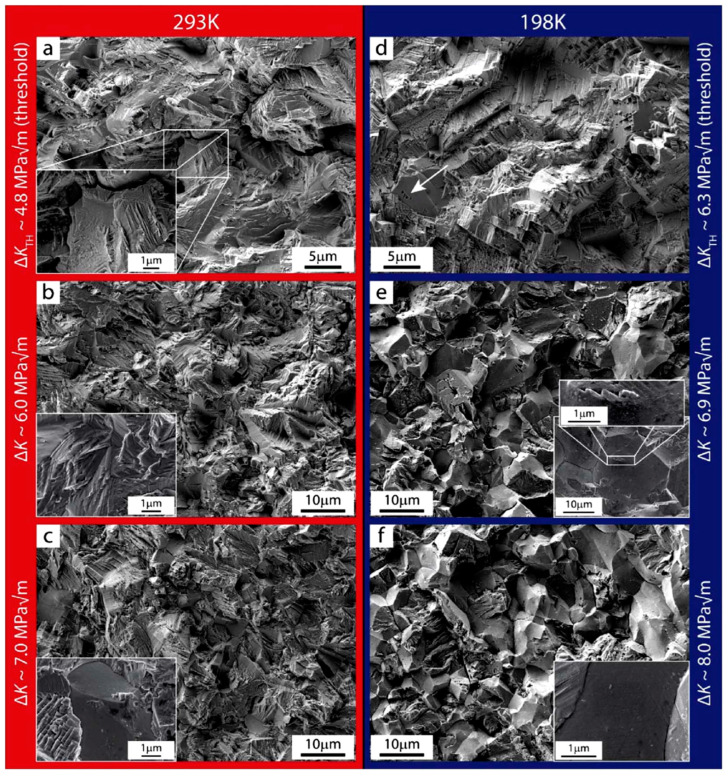
Fracture morphologies of CrMnFeCoNi alloy at 293 K and 198 K. (**a**–**c**) indicates that the fracture surface features of the test sample at 293 K are mainly intergranular crack propagation, the inset of (**c**) shows some areas of slight intergranular crack propagation; (**d**) is a feature of a highly serrated section resulting from a plane slip mechanism, (**e**,**f**) indicates the crack extension in the form of intergranular fracture at higher growth rates [52].

Figure 9 presents the fracture surfaces of specimens at temperatures of 293 K and 198 K. SEM images of the fractured surface of the fatigue sample at 293 K are depicted in Figure 9a–c, revealing transgranular crack propagation. Figure 9d–f illustrates the fracture pattern of a fatigue sample at 198 K. In particular, Figure 9d showcases a sharp, serrated fracture at the edge caused by plane slip. The scanning results at 198 K are also presented in Figure 9e,f, indicating transgranular fracture as the dominant mode of crack propagation. Based on the characterization results, it can be concluded that fatigue-crack propagation at room temperature primarily involves transgranular fracture. Physical contact between mating crack surfaces induces plastic deformation of grains near the crack, thereby reducing the contact area of adjacent fracture surfaces. Consequently, the fatigue fracture at room temperature exhibits a highly pronounced serrated feature along the entire crack path. Conversely, intergranular fracture represents the main crack propagation mode at low temperatures, with a lack of noticeable physical contact between adjacent crack surfaces. This absence of contact intensifies fatigue crack closure caused by roughness.

### 3.2. Effect of Microstructure on Fatigue Crack Growth Behavior

In the study by Liu et al. [62], the outstanding fatigue resistance of a fine-grained Fe_38.5_Mn_20_Co_20_Cr15_Si_5Cu_1.5_ alloy was verified. They employed a combination of fine-grained structure and metastable matrix to successfully overcome the issue of delayed crack initiation but faster propagation rates along grain boundaries in the high-cycle fatigue mechanism of fine-grained materials. The design approach is based on delayed *γ*-*ε* transformation, which involves transforming the crystal structure from a face-centered cubic to a close-packed hexagonal structure. By controlling the phase stability through this phase transformation, the alloy’s stability is maintained during work hardening, and the propagation of fatigue cracks is suppressed. Additionally, the researchers also confirmed the significant role of the ultrafine-grained (UFG) structure in controlling fatigue crack propagation. The Fe_38.5_Mn_20_Co_20_Cr_15_Si_5_Cu_1.5_ alloy underwent friction stir processing to improve its mechanical properties. Subsequently, a room-temperature tensile test was conducted on the processed alloy at an initial strain rate of 10^−3^ s^−1^. The fatigue test utilized a loading frequency of 20 Hz and a stress ratio (*R*) of −1.

**Figure 10:**
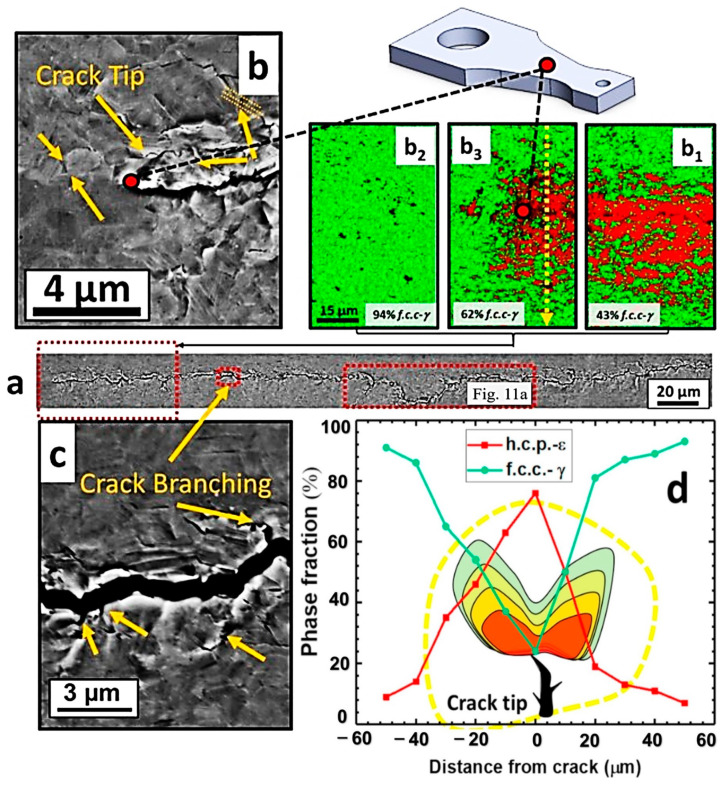
Microscopic characterization of intermittent fatigue experiment, (**a**) overall fatigue crack paths, (**b**) high magnification BSE image of the crack tip, (**b_1_**–**b_3_**) evolution of the crack tip phase due to stress concentration, (**c**) BSE images of cracked branches due to energy depletion, (**d**) phase evolution of the plastic zone at the crack tip [62].

After 114,710 cycles under 717 MPa (0.64UTS), the fatigue test was interrupted to perform microscopic characterization. Figure 10 displays the backscattered electron (BSE) and electron backscatter diffraction (EBSD) images of various regions near the crack tip. The red area in the image indicates a higher volume fraction of *ε* in the plastic region near the crack tip, which also demonstrates the presence of the transformation-induced plasticity (TRIP) effect. However, we need to note that the TRIP effect occurs only if the *γ*-phase is sufficiently stable [50]. After crack initiation, a plastic zone appears at the crack tip, altering the local stress distribution. When the stress concentration in the plastic region reaches a certain level, the TRIP (Transformation Induced Plasticity) effect is triggered. Figure 10b_1_–b_3_,d demonstrate that in the absence of plastic concentration, the phase transformation will not be activated outside the plastic region, which provides evidence for the sufficient stability of the *γ*-phase. In Figure 10c, microcrack branching is observed along the path of crack propagation, leading to increased energy consumption compared to the main crack path. This phenomenon helps to mitigate sudden fatigue failure to some extent. Studies by Liu et al. have also demonstrated that crack retardation can be achieved by employing ultrafine grains and metastable alloys [60,63,64]. However, for materials with short crack propagation paths, faster fatigue failure is observed. To address this issue, researchers employ local work hardening behavior to induce strain mismatch between the two phases or between the matrix and the dispersion. This alteration ultimately modifies the mean free path for crack movement.

**Figure 11:**
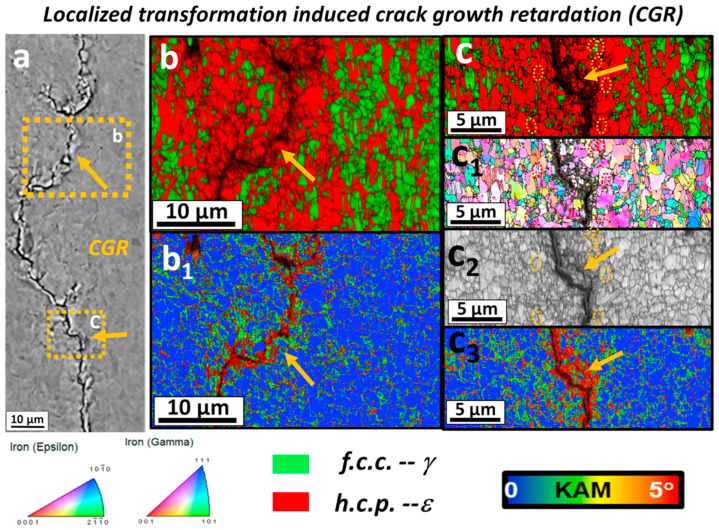
Microscopic characterization of crack growth retardation induced by local deformation. (**a**) is the BSE image of the crack area. (**b**) is the EBSD phase diagram after amplification of region b in (**a**), (**b_1_**) is the KAM analysis of (**b**), (**c**,**c_1_**–**c_3_**) is the EBSD phase diagram, IPF diagram, image quality map, and KAM analysis diagram of region c, respectively [62].

Figure 11a,b depict the occurrence of a local transformation from the *γ* phase to the *ε* phase within the plastic deformation region near the fatigue crack tip. This transformation triggers the formation of microcracks on the primary crack and delays crack initiation. These microcracks disperse the energy associated with the propagation of the primary crack, leading to an increased propagation path. Consequently, fatigue failure is delayed. To sustain the local work hardening activity even under cyclic loading conditions, strain facilitates the dynamic load transfer between the *γ* and *ε* phases. Furthermore, significant cyclic plastic deformation occurs during crack propagation, resulting in additional energy dissipation [65].

In this paper [62], the microstructure of ultrafine grains is firstly used to resist the activation of lattice dislocations and delay the initiation of cracks by reducing the grain size, and then the phase stability is regulated. The strain-induced phase transition is introduced in the plastic region in front of the crack tip to increase energy absorption and delay the transformation from the FCC phase to the HCP phase. The phase transition will cause the microcracks to stay away from the main path, prolong the main crack path, and achieve continuous work hardening to improve the crack initiation and propagation resistance of fatigue cracks, thus hindering the propagation of fatigue cracks. The results verify the possibility that the combination of ultrafine grain and transformation induced plasticity (TRIP) can increase fatigue resistance, and the continuous work hardening activity enhances the material locally in the crack plastic region, thus verifying that the combination of ultrafine grain and TRIP is an effective way to design the next generation of anti-fatigue alloys. The theoretical significance of this study is to reveal the mechanism of the combination of ultrafine grain and metastable alloy on crack retardation, which provides a new idea for material design and fatigue failure control. In a practical sense, the research provides potential methods and strategies for the development of more durable materials and structures, which helps to improve the reliability and life of materials.

Shams et al. [66] investigated the influence of grain size on the low-cycle fatigue behavior of carbon-containing CoCrFeMnNi alloys. Specifically, the researchers aimed to examine the effect of grain size on the tensile and cyclic deformation behavior. Different microstructures were produced through hot-rolling and cold-rolling treatments. The researchers emphasize the importance of forming a fine-grained microstructure without the precipitation of coarse carbides during heat treatment to improve the fatigue life of the material. 

**Figure 12:**
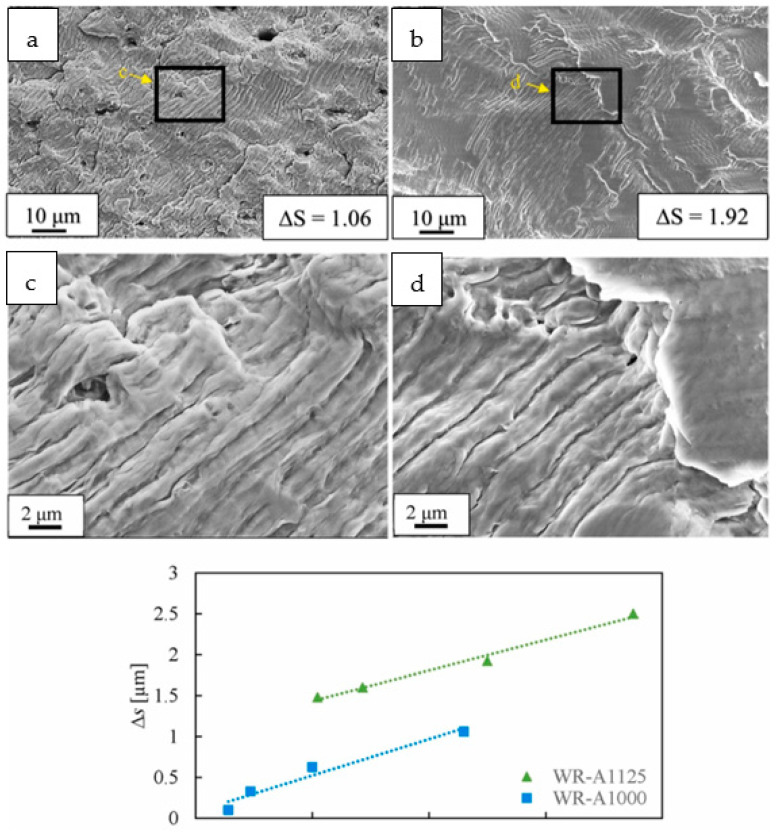
Microscopic characterization of fatigue fracture surface and variation of average crack spacing. (**a**) is the fatigue fracture surface characterization of sample WR-A1000, (**b**) is the fatigue fracture surface characterization of sample WR-A-1125, (**c**) is the high magnification figure of (**a**), (**d**) is the high magnification figure of (**b**). The variation of the average fatigue crack spacing (Δs) of the samples WR-A-1000 and WR-A-1125 with the distance of the fatigue crack nucleation position (L) was also compared [66], The black line area indicated by the yellow arrow is the overall morphology of the fatigue striation enlarged image.

Figure 12 compares the fatigue crack growth behavior at the strain amplitude ($\frac{{\Delta \varepsilon }_{t}}{2}$ = 0.85%) of a fine-grained sample (WR-A1000, *d*~10 μm, A = annealed, WR = warm-rolled) heat-treated at 1000 °C, to a coarse-grained sample (WR-A1125, *d*~66 μm), heat-treated at 1125 °C. It can be observed that reducing the grain size (*d*) from 66 μm to 10 μm leads to a decrease in the average striation pacing (Δs) from 1.92 μm to 1.06 μm when observing the fracture surface at a distance of 500 μm from the crack initiation point (L). This observation suggests that a decrease in grain size effectively retards the growth rate of fatigue cracks. The fine-grain specimen exhibits superior strength and enhanced elastic resistance in comparison to the coarse-grain specimen. This implies that controlling the microstructure can yield positive effects on the initiation and propagation of fatigue cracks. Several studies have provided evidence that reducing the grain size can enhance deformation homogeneity, suppress slip cracking, and decrease the accumulation of slip bands, consequently delaying the initiation and propagation of fatigue cracks [7,9,67].

## 4. Predictive Models for Fatigue Life

Understanding the relationship between fatigue life and applied stress is of utmost importance in the field of reliability design [68]. This section aims to introduce appropriate models and data analysis methods to elucidate this relationship. Typically, fatigue data are represented as an *S-N* curve, which depicts the logarithmic correlation between cyclic stress or strain and the median fatigue life represented in cycles until failure. An extension of this concept is the *S-N-P* curve, which generalizes the relationship between the *P* quantile of fatigue life and applied stress or strain, with each curve reflecting a constant failure probability *P* as a function of *S*. Certain high-entropy alloys exhibit a fatigue limit or endurance limit, which signifies a stress level below which fatigue failure is improbable. The study of infinite fatigue life limit is an active research field, the research focuses on factors such as crack location, direction, size, number, and initiation, which contribute to the inherent randomness of the fatigue limit. When predicting fatigue life, two essential considerations are the observed decrease in standard deviation with increasing stress and the presence of a fatigue limit, as indicated by the curvature of the fatigue curve [14]. In the following section, we will discuss the prediction models for fatigue behavior, considering their application in reliability design.

1. Weibull predictive model

Hemphill et al. studied AlCoCrCuFeNi and used the Weibull predictive model to quantify its fatigue behavior [69]. This model is based on the commonly used analytical expression of the *S-N* curve given by Equation (1). The assumption is made that a Weibull distribution can describe the fatigue life distribution within each fixed stress range.

(1) $$N\left(S\right)={cS}^{-d}$$

where S represents the applied stress range, N(S) is the number of expected fatigue life cycles under S, *c* and *d* are the positive material parameters. Take a natural logarithm of N(S):

(2) $$\log(N(S))=\gamma_{0}+{\;\gamma }_{1}\log(S)$$

where $\gamma_{0}=\log(c)\;\mathrm{and}\;\gamma_{1}=-d$. Equations (1) and (2) describe the relationship between applied stress and the number of cycles until fatigue failure in a test project. However, these equations do not account for the variability observed in actual fatigue life data. To address this issue, an error term ε is introduced to the equations, resulting in a modified equation:

(3) $$\log\left(N\left(S\right)\right)=\gamma_{0}+{\;\gamma }_{1}\log\left(S\right)+\varepsilon$$

Assuming that the error term ε follows the standardized smallest-value distribution, Equation (3) can be transformed into a Weibull regression model. Alternatively, they can be transformed into an equivalent Weibull-accelerated life test model, which has been verified and applied in practical engineering reliability analysis [70]. The fatigue life of the material follows a Weibull distribution at a given stress level *S*, with its probability density function (PDF) and cumulative distribution function (CDF) described by Equations (4) and (5), respectively.

(4) $$f(N(S)|\alpha (S),\beta )=\frac{\beta }{\alpha (S)}{\left(\frac{N(S)}{\alpha (S)}\right)}^{\beta -1}\exp\left(-{\left(\frac{N(S)}{\alpha (S)}\right)}^{\beta }\right)$$

(5) $$F(N(S)|\alpha (S),\beta )\;=1-\exp\left(-{\left(\frac{N(S)}{\alpha (S)}\right)}^{\beta }\right)$$

where β is the Weibull-shape parameter, α(S) is the Weibull-scale parameter that follows $\log\left(N\left(S\right)\right)={\;\gamma }_{0}+{\;\gamma }_{1}\log\left(S\right)$.

To account for censored observations in a four-point bending fatigue test, the Weibull model is used. Censored observation occurs when no fatigue failure is observed after a certain number of loading cycles (10^7^ cycles). The probability of censored observation at a given stress range *S* is determined using Equation (6), which is described below.

(6) $$P\left(N\left(S\right)\geq N_{C}\right)=1-F\left(N_{C}|\alpha (S),\beta \right)=\exp\left(-{\left(\frac{N(S)}{\alpha \left(S\right)}\right)}^{\beta }\right)$$

where $N_{C}$ represents the censor time of the experiment, approximately 10^7^ cycles. The parameters $\gamma_{0}$,$\gamma_{1}$ and β in this equation can be estimated using the maximum likelihood estimation model [69]. The fatigue-experiment data are represented as {(*N_i_*, *S_i_*, *d_i_*) *i* = 1, 2, *m*} [69].

(7) $$L\left(\gamma_{0},\gamma_{1},\beta \right)={\prod_{i=1}^{m}\left[\frac{\beta }{e^{\gamma 0+\gamma 1\log(s_{i})}}{\left(\frac{N_{i}}{e^{\gamma 0+\gamma 1\log(s_{i})}}\right)}^{\beta -1}\right]}^{\delta_{i}}\times \exp\left[-{\left(\frac{N_{i}}{e^{\gamma_{0}+\gamma_{1}\log(s_{i})}}\right)}^{\beta }\right]$$

where *m* is the total number of tested samples, *N_i_* represents the cycle at which failure occurred or was censored, *S_i_* represents the stress imposed on the *i*th sample. If the sample is considered as a truncated observation, *δ_i_* = 0 indicates censoring, while *δ_i_* = 1 indicates non-censoring. Upon completing the parameter estimation, we can predict the fatigue life behavior S at a given stress ratio by estimating the p quantile life. The p quantile life is derived using Equation (8), as described below.

(8) NpS=exp⁡γ0+γ1log⁡S−log⁡1−p1β

**Figure 13:**
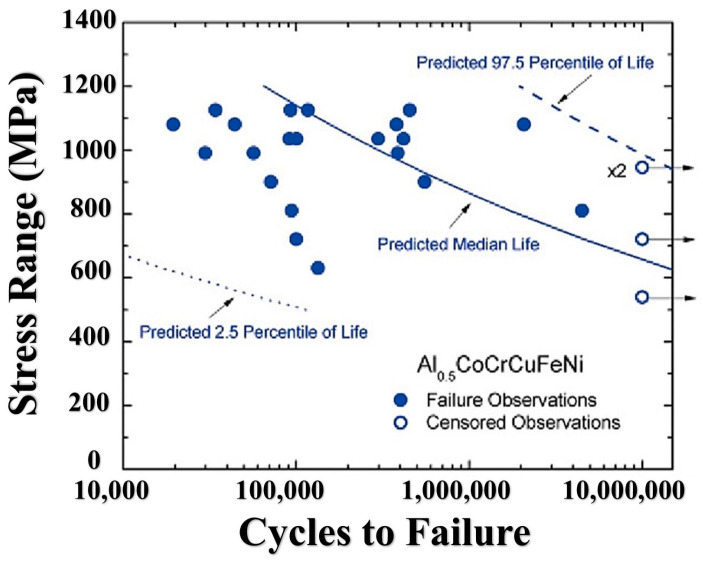
Predicted quantile lives using the Weibull predictive model [69].

The median fatigue life (i.e., = 0.5) is used to describe the relationship between applied stress and average fatigue life response [69]. Additionally, the 0.025 and 0.975 quantiles can be used to construct a 95% interval prediction for constructive fatigue life and quantify the dispersion of fatigue life cycles. In Figure 13, the estimated median fatigue life is depicted, falling within the range defined by the 0.025 and 0.975 quantiles. All the observed failures fall within the 95% predictive interval in this model.

The Weibull predictive model is a widely employed approach for fatigue life prediction in metal materials. This model assumes that the distribution of fatigue life within a fixed stress range can be accurately described by the Weibull distribution. It has been extensively applied to a range of materials, including high-entropy alloys. Moreover, the model effectively handles censored observations in fatigue tests, where no failures are observed within a specific number of loading cycles. Additionally, the model allows the estimation of parameters such as the Weibull-scale parameter and enables fatigue life predictions under specific stress ratios. Nevertheless, as with any statistical model, the Weibull predictive model possesses its limitations and may not fully capture all aspects of material fatigue behavior. Thus, a careful evaluation of the model’s assumptions and restrictions is imperative before its application in practical engineering scenarios.

2. Weibull mixture predictive model

The Weibull predictive model is not always capable of accurately predicting all types of fatigue data, particularly when the data exhibit excessive variability. To address this issue, Hemphill et al. proposed the use of a Weibull mixture prediction model in data processing and analysis [9]. This integrated model consists of two Weibull predictive models, with the fatigue data being separated into strong and weak groups. In specific cases, the variability of the data was assumed to be related to defect density in high-entropy alloy fatigue samples, particularly when the applied stress is less than 1000 MPa. The fatigue life of the weak group was found to be much shorter than that of the strong group. Additionally, when the population of units is non-homogeneous, the Weibull mixture model can be used. This prediction model combines the fatigue life of each stress range value using two Weibull distributions. The PDF and CDF of the Weibull mixture model are given by Equations (9) and (10), respectively.

(9) $$f\;\left(N(s)\;|P,\alpha_{w}\left(S\right),\beta_{w},\alpha_{s}\left(S\right),\beta_{S}\right)\;=\;p\frac{\beta_{w}}{\alpha_{w}\left(S\right)}\times {\left(\frac{N\left(S\right)}{\alpha_{w}\left(S\right)}\right)}^{\beta_{w}-1}\exp\left[{-\left(\frac{N\left(S\right)}{\alpha_{w}\left(S\right)}\right)}^{\beta_{w}}\right]+(1-p)\;\times \;\frac{\beta_{s}}{\alpha_{s}\left(S\right)}{\left(\frac{N\left(S\right)}{\alpha_{s}\left(S\right)}\right)}^{\beta_{s}-1}\exp\left(-{\left[\frac{N\left(S\right)}{\alpha_{s}\left(S\right)}\right]}^{\beta_{s}}\right)$$

(10) $$F\left(N\left(S\right)|P,\alpha_{w}\left(S\right),\beta_{w},\alpha_{s}\left(S\right),\beta_{S}\right)=p\left[1-\exp\left(-{\left(\frac{N\left(S\right)}{\alpha_{w}\left(S\right)}\right)}^{\beta_{w}}\right)\right]+(1-p)\left[\exp\left(-{\left(\frac{N\left(S\right)}{\alpha_{s}\left(S\right)}\right)}^{\beta_{s}}\right)\right]$$

In the Weibull mixture model, the weak and strong groups are represented by *w* and *s*, respectively. The *p*-quantile fatigue lives of the weak and strong groups can be obtained using Equations (11) and (12), which apply the maximum likelihood method similar to that used in the Weibull predictive model.

(11) Np,wS=expγw,0+γw,1logS−log(1−p)1βw

(12) Np,sS=expγs,0+γs,1logS−log(1−p)1βw

**Figure 14:**
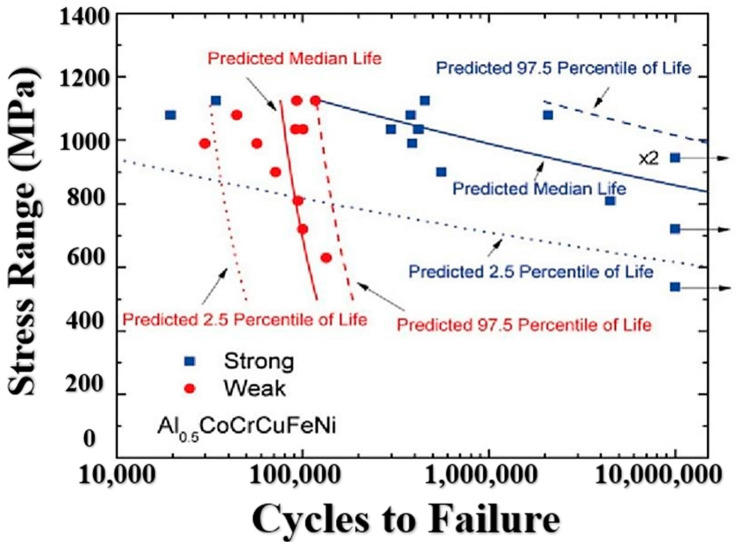
Predicted quantile lives using the Weibull mixture predictive model. Squares are the observations of the weak group; circles are the observations of the strong group [69].

**Figure 15:**
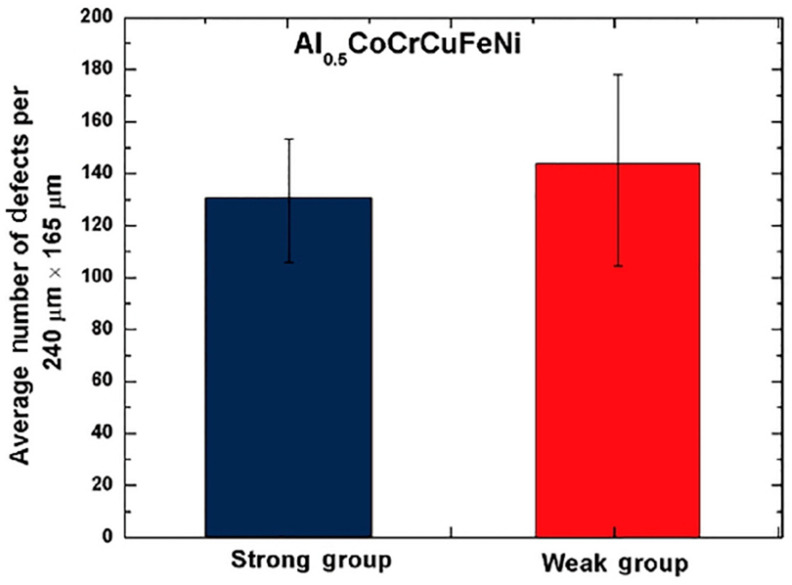
The column charts display the average defect counts for both the strong and weak groups [69].

Figure 14 shows the predicted quantile lives of the Weibull mixture predictive models. The data points are divided into two groups: the weak group, represented by circle dots, described by Equation (11), and the strong group, represented by squares, described by Equation (12). In Figure 15, it is observed that the strong group tends to have fewer defects compared to the weak group, which aligns with the calculations from the Weibull mixture model [22]. The samples with fewer defects exhibit longer fatigue life under cyclic loading.

The model’s ability to capture the heterogeneity of fatigue data makes it a useful tool for analyzing complex fatigue behaviors. However, it requires careful parameter estimation and may be computationally intensive. Furthermore, its underlying assumptions may not always hold in certain scenarios, and as such, its use should be evaluated on a case-by-case basis. Overall, the Weibull mixture predictive model is a valuable addition to the field of fatigue analysis and has been successfully applied in practical engineering applications.

The Weibull predictive model assumes that the fatigue data follow a Weibull distribution, which describes the probability of failure as a function of stress or the number of cycles. This model is efficient for analyzing fatigue data that conform well to a single distribution, without significant variations or subpopulations within the dataset. It provides a simple and straightforward approach for estimating the fatigue life and assessing the reliability of materials. On the other hand, the Weibull mixture predictive model is more suitable for analyzing fatigue data that contain multiple subpopulations with different failure characteristics. This model considers a mixture of Weibull distributions to capture variations in fatigue behavior within the dataset. By identifying and characterizing different subpopulations, it provides a more comprehensive understanding of fatigue phenomena, particularly when the data exhibit heterogeneity. In conclusion, the efficiency of determining fatigue data, using either the Weibull predictive model or the Weibull mixture predictive model, depends on the complexity and heterogeneity of the data. Each model has its strengths and is more appropriate for different types of fatigue data.

## 5. Conclusions and Suggestions

This paper reviews most articles on the fatigue behavior of high-entropy alloys in recent years, The review encompasses a wide range of topics, including fatigue crack initiation, fatigue crack propagation, influential factors, and fatigue life prediction models. Now the conclusion is summarized as follows.

1. Slip is one of the main mechanisms of crack initiation in the CrCoFeMnNi alloy. When deformation occurs on the slip plane created by the slip line, the compactness of the slip line and the planarity of dislocation result in localized slip. Once slip deformation initiates, it tends to concentrate on a single slip surface, leading to the formation of cracks on the slip surface and ultimately causing fatigue crack initiation.
2. By modifying the microstructure, such as introducing new phases and refining grains, it is possible to enhance the fatigue properties of high-entropy alloys, thereby extending the materials’ fatigue life.
3. The fatigue performance of high-entropy alloys is significantly influenced by both inherent defects within the alloy and defects introduced during processing, including micro-voids and surface roughness. Existing research suggests that alloys with fewer defects exhibit improved fatigue properties.

As a novel class of alloys, high-entropy alloys demonstrate superior properties compared to traditional alloys, positioning them as promising candidates for high-strength energetic structural materials. However, investigations into the fatigue behavior of high-entropy-alloy energetic structural materials are still in their early stages. Meeting the growing material demands of modern society necessitates the exploration of high-entropy alloys due to their ability to integrate diverse material properties. Additionally, previous studies have confirmed the potential applications of high-entropy alloys in terms of fatigue behavior. Therefore, it is crucial to delve deeper into the understanding of their fatigue behavior through the following proposed research tasks.

1. The fatigue strength of high-entropy alloys under various loading conditions should be systematically studied. This investigation should encompass fatigue crack initiation, propagation, and final failure analysis. It is important to identify the critical factors influencing fatigue behavior, such as microstructural characteristics, alloying elements, and processing techniques.
2. A comprehensive understanding of the effect of environmental conditions on fatigue performance is necessary. This includes evaluating the impact of temperature, humidity, and corrosion on the fatigue behavior. The interaction between environmental factors and microstructural evolution should also be investigated to determine their influence on fatigue life.
3. It is essential to explore the influence of different heat treatments and thermomechanical processing techniques on fatigue behavior. Optimized heat treatment methods can potentially improve the fatigue life and mechanical properties of high-entropy alloys. Therefore, investigating the microstructural evolution and mechanical response subjected to various thermal and mechanical treatments is crucial.
4. Developing reliable and accurate predictive models for the fatigue life is of great importance. Utilizing advanced computational methods, such as finite element analysis and machine learning algorithms, can aid in establishing a comprehensive understanding of the fatigue behavior of HEAs. These models can facilitate the design and optimization of high-entropy alloy structural materials for specific applications.

## Figures and Tables

**Table 1:** Comparison of fatigue crack expansion parameters of high-entropy alloys.

Composition	Temperature	Paris Slope (*m)*	Frequency (Hz)	Δ*K_th_* (MPa√m)
Zr_41.2_Ti_3.8_Cu_12.5_Ni_10_Be_22.5_	RT	2.7	25	3
CrMnFeCoNi HEA	198 K	4.5	25	6.3
CrMnFeCoNi HEA	RT	3.5	25	4.8
Al_0.2_CrFeNiCu_0.2_ HEA	RT	4.9	20	16
AlCrFeNi_2_Cu HEA	RT	3.4	20	17

RT = Room Temperature.

## Data Availability

The data presented in this study are available on request from the corresponding author.
